# Interactions of LC8 with N-Terminal Segments of the Intermediate Chain of Cytoplasmic Dynein

**DOI:** 10.1100/tsw.2003.56

**Published:** 2003-08-02

**Authors:** Afua Nyarko, Michael Hare, Moses Makokha, Elisar Barbar

**Affiliations:** Department of Chemistry and Biochemistry, Ohio University, Athens, Ohio 45701, USA

**Keywords:** Macromolecular assembly, Dynein light and intermediate chains, conformational change, limited proteolysis

## Abstract

LC8, a highly conserved 10-kDa light chain, and IC74, a 74-kDa intermediate chain, are presumed to promote the assembly of the cytoplasmic dynein motor protein complex and to be engaged in the controlled binding and release of cargo. The interactions of LC8 from Drosophila melanogaster with constructs of IC74 were characterized in vitro by affinity methods, limited proteolysis, and circular dichroism spectroscopy. Previously, we have performed limited proteolysis on the N-terminal domain of IC74, IC(1-289), when free and when bound to dynein light chains LC8 and Tctex-1[1]. We have also shown that upon addition of LC8, IC(1-289) undergoes a significant conformational change from a largely unfolded to a more ordered structure. The purpose of the work presented here is to determine whether residues 1-30 in IC74, predicted to be in a coiled coil, are involved in the stabilization of the protein upon binding to LC8. Constructs of IC74, IC(1-143), and IC(30-143) that include the LC8 binding site but with and without the first 30 residues were prepared, and their binding and protection patterns were compared to our previous results for IC(1-289). The results suggest that coiled coil residues 1-30 are not responsible for the increase in structure we observe when IC(1-289) binds to LC8.

### INTRODUCTION

Cytoplasmic dynein is an essential participant in chromosome movement during mitosis, in the trafficking of vesicular cargo, and in positioning various organelles during the interphase. It is a

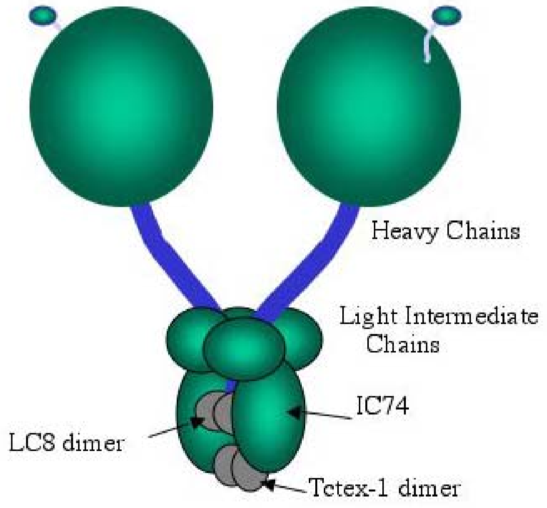

**FIGURE 1**. Schematic representation showing the relationship of the components of dynein to each other. This relation of the motor head and stalk domains to the light and intermediate subunits is based on images from electron microscopy[25]. The complex contains a number of light chains in addition to LC8 and Tctex-1. LC8 and Tctex-1 are both dimers at physiological conditions as determined from our work and others[26,27,28].

principal motor for motion along microtubules, toward their minus end. Cytoplasmic dynein is a large (1.2 MDa) protein complex made up of two heavy chain subunits linked by stalk-like domains to a common base (Fig. 1). The heavy chains contain the ATP-hydrolysis and microtubule binding sites required for motility. Located at the base are a number of subunits thought to regulate the assembly of the complex and its attachment to appropriate cargo[2].

The intermediate chain IC74 forms a key intermediary in the complex, as it associates with the heavy chain and the accessory complex dynactin as well as with other subunits in the base[3,4]. IC74 is comprised of two independently folding domains as predicted from secondary structure analysis. The N-terminal domain, IC(1-289), is predicted to be disordered except for two short segments of coiled coil, 1-30, and 209-239. The C-terminal domain IC(290-643) is predicted to be predominantly β-sheet with seven WD repeats folded into a β-propeller. Truncation mutations of these domains show that they have independent functions: the N-terminal domain binds dynactin, while the C-terminal domain binds the heavy chain[5]. We have shown by circular dichroism, fluorescence spectroscopy, sedimentation velocity, and the relative ease of proteolysis that IC(1-289) is predominantly unfolded with elongated noncompact structure[1]. Far UV CD, however, indicates the presence of a small percentage of helical or coiled-coil structure, consistent with coiled coil.

All known cytoplasmic and axonemal dyneins as well as myosin V contain LC8[6,7,8,9,10]. LC8 has an unusually high degree of sequence homology: *Drosophila* LC8 is 94, 71 and 50% identical with LC8 from human, *Aspergillus nidulans*, and yeast, respectively. Mol. genetic studies in several organisms including *Drosophila*, *Chlamydomonas*, and *Aspergillus* show the functional significance of this subunit in oogenesis and female sterility, nuclear migration and positioning, and flagellar motility and retrograde intraflagellar transport[8,12,13].

We have found that LC8, as well as the dynein light chain Tctex-1, attach to the N-terminal domain of IC74, IC(1-289). Using limited proteolysis followed by mass spectrometry, we have further localized the sequence necessary for binding the light chains to residues 99-147[1]. In these experiments, retention of the native shape is favored since the 289-residue domain is large and proteolysis is administered under near-physiological conditions. A most interesting observation from these studies was that IC(1-289) appears to gain stability and secondary structure upon binding to LC8, as evidenced by an increase in CD-detected structure and generalized resistance to proteolysis. In order to determine whether the N-terminal fragment (with residues 1-30) predicted to be coiled-coil, contributes to the stabilization of IC74, we have prepared smaller constructs of IC74, IC(1-143), and IC(30-143), and assayed them for binding and protection.

### METHODS

#### Cloning, Overexpression, and Purification of IC74 Constructs

For preparation of IC74 constructs, wild type cDNA (accession # AF26337) was used as a template. Synthesis of the N-terminal sequences 1-289, IC(1-289), 1-143, IC(1-143), and 30-143, IC(30-143), were achieved by PCR amplification with BamH1 and HindIII restriction sites. The constructs were ligated into pET 15d plasmid, and transformed into BL21(DE3) (Novagen) bacterial expression cell lines. Preparation of IC(1-289) was accomplished as published[1]. The constructs IC(1-143) and IC(30-143) were prepared as follows. The recombinant BL21(DE3) cells were inoculated in a 1-liter LB medium containing ampicillin (60 μg/ml) from an overnight culture and grown at 37°C to an OD600 of 0.6 to 0.8. IPTG was added to a final concentration of 0.1 m*M* and the culture was incubated at 27°C for another 4 h. The cells were harvested by centrifugation at 4000 g for 30 min and resuspended into 40 ml of 0.1 *M* sodium phosphate buffer pH 8 containing 5 m*M* β-mercaptoethanol, 1 m*M* PMSF, and 0.1 mg/ml lysozyme then lysed by sonication. Crude extract after centrifugation was purified by affinity chromatography on Ni-NTA resin (Qiagen) by elution in 50 m*M* imidazole buffer. Purity of >95% was confirmed by SDS-PAGE.

#### GST Pull-down Assays

GST-LC8 was prepared by inserting LC8 into BamH1 and SalI restriction sites in the pGEX-6p-3 plasmid (Amersham Pharmacia Biotech). Sepharose precipitation of IC constructs with GST-tagged LC8 was used to screen for binding. GST-LC8 was added to a slurry of GST-Sepharose 4B resin (Amersham Pharmacia Biotech) in phosphate buffer saline, and the suspension was agitated at 4˚C for 30 min. The GST-LC8 bound beads were washed three times in PBS buffer and 1% Tween to remove any unbound protein. An equimolar concentration of IC constructs were added and the suspension agitated at 4˚C for an additional 30 min. The resin was recovered by centrifugation and after excessive washing, samples were resuspended in electrophoresis sample buffer, analyzed on a 15% SDS-PAGE gel, and visualized by Coomassie Brilliant Blue staining.

#### Limited Proteolysis

Limited proteolysis experiments were carried out using the nonspecific enzyme proteinase K (Novagen). All reactions were performed in 50 m*M* sodium phosphate buffer, pH 7.8. IC

constructs, IC(1-289), IC(1-143), and IC(30-143) at a concentration of 15 μ*M* were incubated (in separate experiments) for 3 h at 4°C in the presence and absence of equimolar concentration of LC8 in a final reaction volume of 100 μl. The samples were then treated with proteinase K (enzyme substrate ratio 1:500), and incubated at 0°C. The protease concentrations were chosen such that the small differences in the digestion of the free and bound IC constructs were maximized. At 3- to 5-min intervals 10 μl aliquots were removed and treated with the protease inhibitor phenylmethanesulfonyl fluoride (Sigma) to a final concentration of 2 m*M*. Samples were run on a tricine SDS-PAGE gel[14], and visualized by silver staining. Protein substrates and proteases were prepared according to published methods[15].

### Circular Dichroism Spectroscopy

Circular dichroism (CD) spectra were obtained using a JASCO 715 spectropolarimeter. To monitor interactions by CD, the spectrum of a stoichiometric mixture of two proteins (experimental) was compared to the sum of the individual spectra of the proteins (model for no interaction). All experiments were conducted at 25˚C with protein concentrations of 7 μ*M* in a 1-mm path length cell. For the mixing experiment, molar ellipticity was calculated using a protein length and concentration corresponding to the average of the two proteins[16].

## RESULTS

### Increase in Protection, and Global Structure in IC(1-289) upon Binding to LC8

Limited proteolysis using the nonspecific enzyme proteinase K and subsequent analysis on SDS-PAGE identified several high Mol. weight fragments of IC74 that are protected, significantly in the presence of LC8 (Fig. 2). Lanes 5 to 16 show digestion of IC(1-289) in the presence and absence of LC8 at time intervals 1 to 30 min. Two bands indicated by arrows in the Mol. mass range of 23 to 29 kDa are significantly more protected in the presence of LC8 (lanes 7, 9, 11). Another band ~ 20 kDa persists in free IC(1-289) but is more protected with LC8 at longer times (Fig. 2 and data not shown). Fig. 3 shows the difference in CD spectra between an equimolar mixture of LC8 and IC(1-289), referred to as mixture, and the sum of the corresponding spectra, which represents a spectrum for noninteracting proteins or for interactions that are not

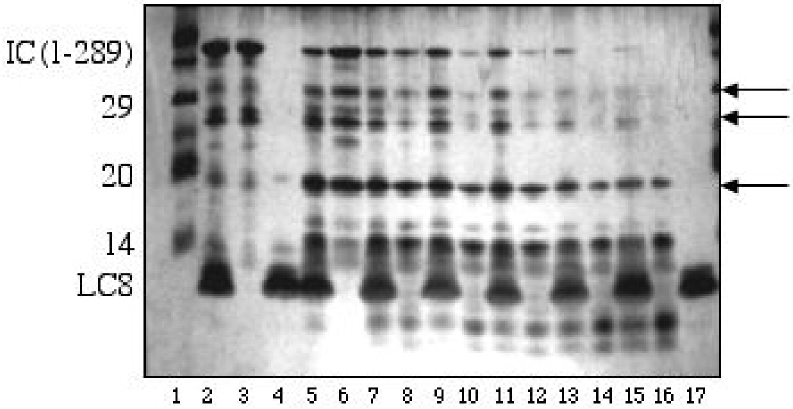

**FIGURE 2.** SDS-PAGE gel showing proteinase K digests of IC(1-289) in the presence (lanes 5, 7, 9, 11, 13, and 15), and absence of LC8 (Lanes 6, 8, 10, 12, 14, and 16) with digestion time of 1, 5, 10, 15, 30 and

60 min. Lane 2 is mixture of LC8 and IC(1-289) at time 0. Lanes 3 and 4 are free IC(1-289), and LC8 at time 0, respectively. Lane 17 shows 30 min digestion of free LC8 indicating that LC8 is not cleaved under these conditions. Mol. weight markers are shown in lane 1. The large Mol. weight fragments (pointed to by arrows) and that of IC(1-289) persist significantly longer in the presence of LC8, while rapid degradation is observed in the absence of the ligand. Digestion was carried out at 4 ˚C, with an enzyme-substrate ratio of 1:500. Gels were visualized by silver staining.

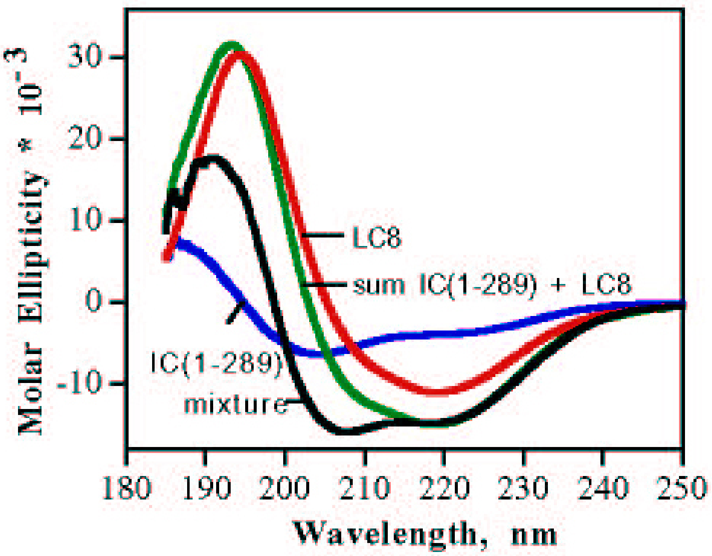

**FIGURE 3.** Changes in CD-detected secondary structure upon interaction of IC(1-289) with LC8 at equimolar concentration shown as molar ellipticity vs. wavelength. Spectra of IC(1-289) are in blue, LC8 in red, the spectra of the mixture in black, and the sum of individual spectra of the two proteins (model for no interaction) in green. A difference between the mixture and the sum indicates a conformational change upon interaction.

accompanied by conformational change. There is a significant increase in negative ellipticity at 208 nm in the mixture relative to the sum indicating an increase in helical or coiled coil structure, presumably in IC(1-289).

### IC(1-143) and IC(30-143) Bind LC8 but Show No Observable Increase in Structure upon Binding

To determine whether the first 30 residues predicted to form a dimeric coiled coil contribute to the increase in helical or coiled coil structure observed by CD upon addition of LC8, we prepared smaller constructs, IC(1-143) and IC(30-143). Both constructs include the sequence necessary for binding to LC8. LC8 binding was demonstrated by GST pull-down assay experiments using GST fused to LC8 (Fig. 4). Lane 3 shows a band for IC(30-143) when GST-LC8 is present. No band is observed with GST, or with the resin indicating that the interaction is specific to IC(30-143) (lanes 2 and 5, respectively). Similar results were obtained for IC(1-143) (data not shown).

Limited proteolysis with proteinase K under conditions similar to proteolysis of IC(1-289) was performed on both constructs (Fig. 5). No protection of the intact constructs or any large fragments was observed in the presence of LC8 suggesting that the sequence of IC that gives rise to protection is most likely downstream of residue 143. Since LC8 is not cleaved at the conditions of the experiment as shown by the absence of smaller bands in lane 17 in Fig. 5A and B, the band corresponding to smaller Mol. weight (below LC8 on the gel) corresponds to a sequence of IC that gets protected due to LC8 binding. CD spectra of interactions of IC(1-143) and IC(30-143) with LC8 show no difference between the mixture and the sum, supporting the protection experiment and indicating that interactions are not accompanied by a detectable change in the secondary structure (data not shown).

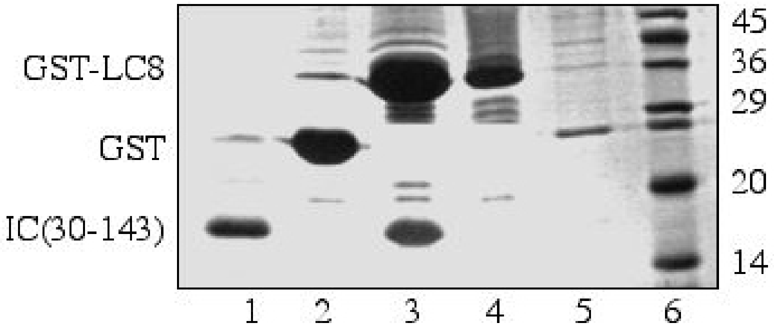

**FIGURE 4.** SDS-PAGE gel showing the interaction of LC8 with IC(30-143) by GST pull down affinity precipitation. Pure IC(30-143) and GST-LC8 are shown in lane 1 and lane 4 respectively. IC(30-143) was retained on the sepharose resin containing GST-LC8 (lane 3) but not with GST alone (lane 2) nor with the resin (lane 5) indicating that binding of IC(30-143) is specific to LC8. The gel was stained with Coomassie Brilliant Blue. Positions of Mol. mass markers in kDa are shown on the right.

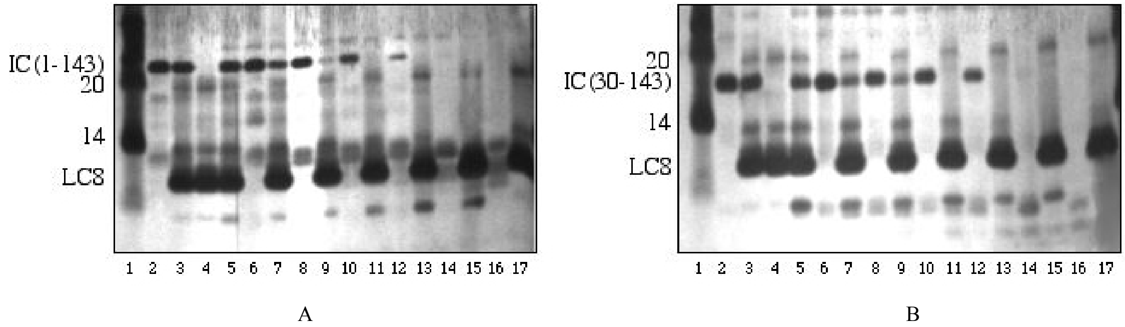

**FIGURE 5.** SDS-PAGE gel showing proteinase K digests of IC(1-143) (A), and IC(30-143) (B) in the presence and absence of LC8. Digestion was carried out at 4 ˚C, with an enzyme-substrate ratio of 1:500 at an equimolar protein concentration of 15 μM. Mol. weight markers are shown in lane 1. Lanes 2 and 4 are free IC(1-143) in A, and IC(30-143) in B, and LC8 at time 0 respectively. Lane 3 is mixture of LC8 and IC constructs at time 0. Lanes 5, 7, 9, 11, 13, and 15 are digests in the presence of LC8, while lanes 6, 8, 10, 12, 14, and 16 are digests in the absence of LC8 with digestion time of 1, 5, 10, 15, 30 and 60 min. Lane 17 shows 30 min digestion of free LC8 indicating that LC8 does not produce any fragments under these conditions. Gels were visualized by silver staining. In contrast to IC(1-289), no protection of intact IC constructs or large fragments is observed in the presence of LC8.

### Residues Remote from the LC8 Binding Site May Contribute to Stabilization

Since several large fragments of IC(1-289) are protected in the presence of LC8, identification of their sequences will ascertain the identity of the residues that contribute to global stability. Purification of these fragments by RP-HPLC for further identification by N-terminal sequencing was not possible due to difficulties in separation. Instead, we have used in-gel digestion. The bands indicated by arrows in Fig. 2, after 10 min of proteinase K digestion, were cut from the gel, digested with trypsin, and the resulting peptides were extracted for identification by mass spectrometry. The larger band consists of fragments that span residues 184-288. The smaller bands contain fragments that span a shorter sequence and include fragments that correspond to residues 205-237. Since the three most protected large Mol. weight bands correspond to sequences that are C-terminal to the LC8 binding site, we suggest that the C-terminal segment of

IC(1-289), which includes a sequence predicted to form a coiled coil 209-239, contributes to the increase in structure. The masses on the gel are lower than predicted from Mol. weight markers because the protein is highly charged and does not bind tightly to SDS. For example, the band at ~ 20 kDa is closer to 13 kDa as determined from mass spectrometry[1].

### DISCUSSION

We have initiated studies to characterize the assembly of three subunits located at the base of cytoplasmic dynein complex: the light chains LC8 and Tctex-1 and the intermediate chain IC74. These subunits interact tightly as a subcomplex within the base of the native dynein complex[17], bind to many unrelated cargoes, and are highly conserved among species. IC74 binds to dynein heavy chain, linking the light chains to the heavy chains, and it also binds to dynactin[3,4], which is proposed to mediate the association of numerous cellular cargoes with the dynein motor and to further facilitate the processive movement of cargo along the microtubule[18,19,20].

We have recently reported that the binding sites for both LC8 and Tctex-1 are in the vicinity of residues 99-147 of IC74[1]. This sequence was identified by limited proteolysis using both specific and nonspecific enzymes. The fragments were purified by HPLC, and identified by mass spectrometry. We have further reported that there is a significant conformational change, presumably in IC(1-289), that is observed upon binding to LC8. The N-terminal domain is predicted to be disordered using PSIPRED, a secondary structure analysis routine[21]. There are two coiled coil segments predicted by MultiCoil[22] for residues 2-30, and residues 209-239. The prediction of coiled coil–forming segments is highly significant, since these structures are frequently found to play a key role in the formation of protein complexes. In this work, we prepared constructs of IC74 and assayed for binding and protection in order to identify the segment(s) on IC74 that is responsible for the increase in structure, and to investigate whether the predicted coiled coil segments play a role in increasing the order of IC(1-289) upon LC8 binding.

A considerable increase in ordered structure in IC(1-289) accompanies binding of LC8, detected as a global increase in protection from proteolysis (Fig. 2) and a clear increase in signal at 208 nm in the circular dichroism spectra (Fig. 3). The change in circular dichroism in the presence of LC8 is consistent with an increase in helical structure in IC(1-289) possibly as the result of greater ordering in one or both of the predicted coiled-coil regions. In contrast, the two smaller constructs, IC(1-143) and IC(30-143), bind to LC8 but do not show any observable increase in structure upon binding, indicating that this part of the sequence does not contribute, on its own, to the observed increase in structure. This conclusion is supported by the identity of the bands on the gel that are protected in the presence of LC8. The sequences of these bands correspond to residues 184-288, and are further cleaved to a stable domain that includes residues 205-237, which correspond to the predicted second coiled coil domain.

Although unstructured proteins of this size are not often reported, the disorder predicted for IC(1-289) is consistent with a recent database that shows a significant proportion of gene sequences code for proteins that are intrinsically disordered rather than folded and globular[23]. Such proteins have varied roles in the cell, and are generally thought to undergo folding transitions upon binding to their cellular targets[24]. It is interesting to note that while the sequence of IC(1-289) is not highly conserved among species (23% homology between *Drosophila* and *Dictyostelium*, 30% with mouse), the disorder predicted from their sequences *is* conserved. These observations suggest that the folding transition of IC74 on binding to LC8 is functionally relevant, and is necessary for the formation of an active dynein complex.

### ACKNOWLEDGMENT

This work was supported by National Institutes of Health Grant GM60969 (to E.B.), and American Cancer Society (to M.H.)
